# The effect of ageing on shear wave elastography muscle stiffness in adults

**DOI:** 10.1007/s40520-019-01139-0

**Published:** 2019-02-14

**Authors:** Abdulrahman M. Alfuraih, Ai Lyn Tan, Philip O’Connor, Paul Emery, Richard J. Wakefield

**Affiliations:** 1 Radiology and Medical Imaging Department, College of Applied Medical Sciences, Prince Sattam bin Abdulaziz University, Kharj, Saudi Arabia; 2 Chapel Allerton Hospital, Leeds Institute of Rheumatic and Musculoskeletal Medicine, University of Leeds, Leeds, UK; 3 NIHR Leeds Biomedical Research Centre, Leeds Teaching Hospitals NHS Trust, Leeds, UK

**Keywords:** Elastography, Muscle, Stiffness, Age, Ageing, Ultrasound

## Abstract

**Background:**

Skeletal muscle undergoes structural changes with ageing which may alter its biomechanical properties. Shear wave elastography (SWE) may detect these changes by measuring muscle stiffness.

**Aims:**

To investigate muscle stiffness in healthy young, middle-aged and elderly cohorts using SWE and correlate it with muscle strength and mass.

**Methods:**

Shear wave velocity (SWV) was measured in the quadriceps, hamstrings and biceps brachii of 26 young (range 20–35 years), 21 middle-aged (40–55) and 30 elderly (77–94) volunteers. The participants performed several muscle tests to evaluate their strength. The One-way ANOVA was used to test the muscle stiffness differences between the groups and the Pearson’s correlation coefficient to evaluate the relationship between SWV and muscle strength.

**Results:**

The overall resting muscle SWV gradually decreased with age but was only significantly reduced in the elderly group (*p* < 0.001); with the exception of the vastus lateralis SWV where a significant difference was noted (*p* < 0.05) between young (1.77 m/s), middle-aged (1.64 m/s) and elderly (1.48 m/s). The elderly group had on average 16.5% lower muscle stiffness compared to the young. SWV significantly correlated with muscle mass (*r* = 0.316), walking time (*r* = − 0.560), number of chair stands (*r* = 0.522), handgrip strength (*r* = 0.436) and isokinetic knee strength (*r* = 0.640). Sex and BMI did not explain any significant variation in SWV.

**Conclusions:**

Ageing was associated with a decline in skeletal muscle stiffness which positively correlates with muscle weakness. Further research is needed to evaluate the promising role of SWE as a biomarker for sarcopenia assessment and potential falls risk prediction in elderly individuals.

**Electronic supplementary material:**

The online version of this article (10.1007/s40520-019-01139-0) contains supplementary material, which is available to authorized users.

## Introduction

It is known that muscle mass decreases with age [1]. Loss of mass and strength in elderly individuals are associated with increased risk of falls and loss of independence [2]. Histologically, ageing is associated with changes in muscle composition including myosteatosis, myofibrosis [3] and dysfunction in extracellular elastic fibres [4]. Such changes may alter the biomechanical properties resulting in changes to muscle stiffness.

Ultrasound shear wave elastography (SWE) is a new quantitative and non-invasive method used for assessing tissue stiffness. It has been widely utilised in breast, liver and thyroid [5]. However, there is minimal data on normative values for muscle stiffness and how it changes with age. SWE may offer new insights into how the ageing phenomenon affects the biomechanical properties of muscle and how it relates to muscle quality aspects. Previous evidence has demonstrated how tendon stiffness is significantly reduced in older populations [6]. However, no research to date has been dedicated to investigate how ageing may influence muscle stiffness using SWE and how it correlates with other primary variables such as muscle mass and strength.

Conventional biomechanical testing on animal models shows that muscle stiffness is not affected by ageing [7]. However, there are several, albeit not consistent, theoretical reasons why muscle stiffness might change with ageing. For example, it has been reported that an increase in collagen fibres in ageing muscle promotes an increase in muscle stiffness [8]. In contrast, others suggest that the elastic fibre system in the muscle extracellular matrix loses some of its resistance property and becomes softer with ageing [9]. Additional factors known to occur in aged muscles such as myosteatosis, variability in fibre sizes and impaired connective tissue support the second hypothesis [10, 11].

The primary aim was to investigate to what extent muscle stiffness measured by SWE differed amongst healthy young, middle-aged and elderly cohorts. The secondary aim was to understand how muscle stiffness correlates with muscle strength and mass in different age groups.

## Methods

### Study design

This study was conducted in a cross-sectional design by recruiting healthy participants from three age groups, young (20–35 years), middle-aged (40–55 years) and elderly (above 75 years), and testing their muscle stiffness, strength, function and body composition in a single visit. Assessments were conducted at the Leeds Biomedical Research Centre based at Chapel Allerton Hospital in Leeds, UK from May 2017 to September 2018.

### Eligibility and recruitment

Participants’ eligibility for the young and middle-aged groups was based on the following:(1) aged 18–35 years old or 40–55 years old, respectively; (2) being asymptomatic; (3) no previous history of musculoskeletal or neurological disorders; (4) not currently taking or previously taken a corticosteroid treatment for the past three years with doses > 5 mg/day; (5) not currently taking or previously taken a HMG-CoA reductase inhibitors (statins) for the past 3 years. The same criteria apply to the elderly group except they may have concomitant osteoarthritis due to the high prevalence of this disease in the elderly population, which can be impossible to exclude.

The elderly participants were invited from a database of a collaborative research study [the Community Ageing Research 75+ (CARE 75+)], which provided the contacts of community-dwelling elderly volunteers aged above 75 who agreed to be approached by other research studies.

To select a healthy elderly cohort, all elderly participants had an Edmonton frailty scale of ≤ 5 (not frail), an English Longitudinal Study of Aging (ELSA) frailty index score of ≤ 14 (‘fit’ and ‘well’) and Montreal Cognitive Assessment (MoCA) score of ≥ 20 (no signs of dementia). Moreover, they were screened to ensure none were sarcopenic according to the European working group on sarcopenia in older people (EWGSOP) criteria [1]. Muscle mass was measured using the Tanita DC-430 MA (Tanita Europe B.V., UK) bioelectrical impedance analysis system. The sarcopenia assessment flowchart is illustrated in Supplementary Fig. 1 highlighting that all elderly subjects were non-sarcopenic.

### Shear wave elastography

SWE was performed using the two-dimensional Aixplorer (Supersonic Imagine, Aix-en-Provence, France) system using the SuperLinear™ SL10–2 MHz probe. The principle of SWE can be found in other reviews [5]. Briefly, the system evaluates tissue stiffness by measuring the propagation velocity of shear waves inside the tissue in meters per second (m/s). The shear wave velocity (SWV) increases proportionally with the elasticity modulus and can be used as a surrogate for tissue stiffness.

The technical acquisition methods were adapted from our previous work [12, 13]. Namely, the ultrasound probe was oriented along the muscle fibres. The region of interest (ROI) size was set at 10 mm. SWE acquisitions were repeated three times per muscle and recorded in units of meters per second (m/s). The probe was placed on top of the skin with a minimal load ensuring no external pressure could affect the measurements.

**Fig. 1:**
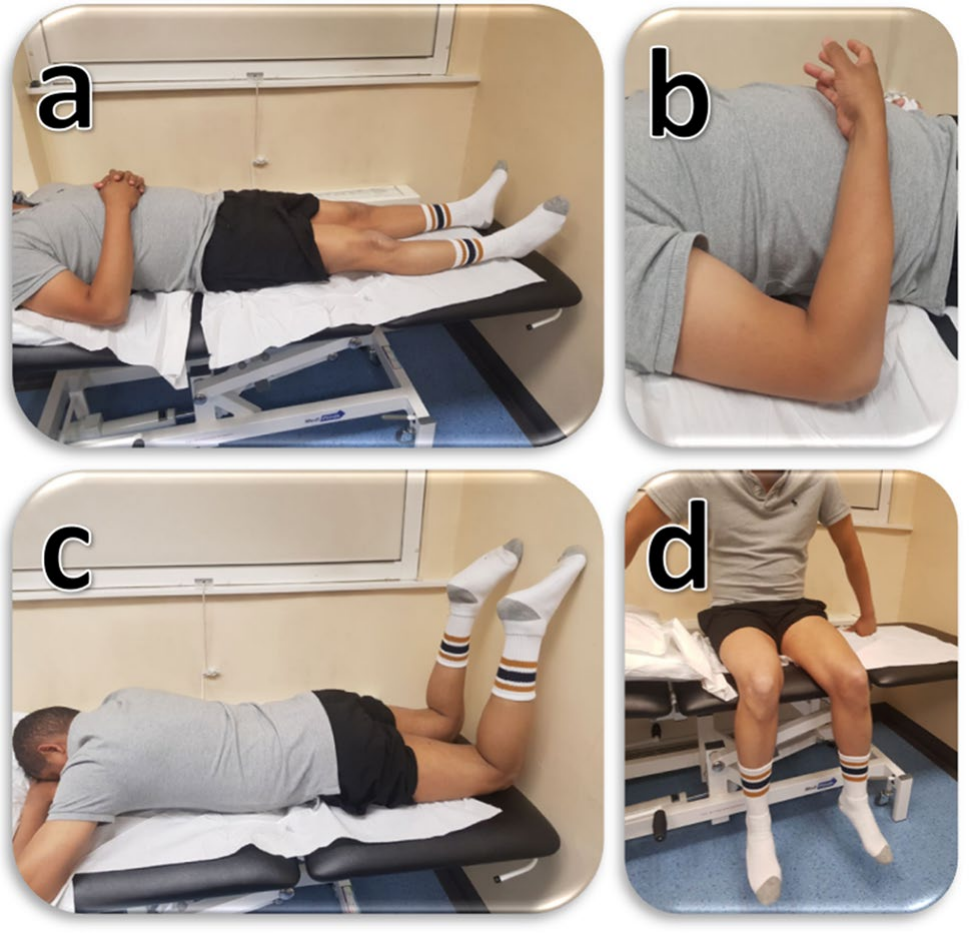
Muscle SWE measurement positions. **a** (quadriceps rested): supine on a flat bed. **b** (biceps brachii rested): elbow flexed at 90° with the forearm rested on the body and hand in supination. **c** (hamstrings rested): prone on a flat bed with the knees flexed at 90° and rested on a wall. **d** (quadriceps passively stretched); seated with the hips and knees flexed at 90° and feet hanging outside the table without touching the floor

The muscles investigated were the four quadriceps [vastus lateralis (VL), rectus femoris (RF), vastus medialis (VM) and vastus intermedius (VI)], the three hamstrings [biceps femoris (BF), semitendinosus (ST) and semimembranosus (SM)] and the biceps brachii (BB). The muscles were scanned in the most relaxed muscle positions (Fig. 1). The quadriceps were also assessed during static passive stretching (90° knee flexion position) to investigate the muscle elastic property when the muscle fibres are elongated under passive tension without any active force or load applied. The other muscles were not tested during static passive stretching to avoid prolonging the total scanning time (30 min). These positions demonstrated reliable readings in our previous work and other studies [12, 13]. The scan was limited to the dominant side since limb dominance had no significant impact on muscle stiffness [12]. Before scanning, all participants were placed in a supine position on the scanning bed and were asked to relax and be comfortable for 5 min. All participants were asked to refrain from any strenuous or sporting activities at least 1 day prior to the study to minimise possible confounding exercise effect.

### Muscle assessments

#### Handgrip strength

Isometric handgrip muscle strength was tested using the Jamar Plus+^Ⓡ^ electronic hand dynamometer (Lafayette Instrument Company, USA). The participants were asked to perform maximal voluntary contraction, and the mean strength of three measurements was recorded for the dominant hand.

#### Expanded timed get-up-and-go (ETGUG)

Participants were asked to stand from a chair (sit to stand) then walk at their normal pace towards a cone placed 9.5 metres away (gait initiation and walk 1), turn around and walk back towards the chair (turn around and walk 2) then sit down (slow, stop, turn and sit). The times to perform these tasks were recorded using a stopwatch.

#### 30-s chair stand test (CST)

Participants were asked to sit in the middle of a standard 43 cm high chair placed against a wall with their arms crossed over chest, feet flat on floor and shoulder width apart before being instructed to perform the maximum number of chair sit to stands they can within 30-s. The total number of sit to stands within the 30-s window was recorded.

#### Knee extension/flexion strength

The Biodex system 4 (IRPS Mediquipe, UK) was used to assess the isokinetic knee extension and flexion strengths. The chosen protocol tested the concentric isokinetic knee extension and flexion movements set at 60°/s angular velocity. The range of motion was set at the maximum that each participant can achieve to obtain maximal speed at the isokinetic motion (usually ranged from 0°–90° to 0°–120°). The participants performed three sets of three knee extension and flexion repetitions in sequence at 100% effort. They had a 30-s rest period between the sets. The mean extension and flexion weight-normalised peak torque [Newton-meters (Nm/kg)] and average power (Watts/kg) were recorded.

### Statistical analysis methodology

The G*Power statistical power analyses software was used to calculate the sample size for the one-way ANOVA test based on the effect size from a relatively similar previous study [14]. At alpha = 0.05, power (1 − Beta) = 0.90, effect size of 0.48 between the three groups, the required sample size is *n* = 60, suggesting at least 20 participants per group.

Statistical analyses were undertaken using SPSS version 25 (Armonk, NY: IBM Corp). The one-way ANOVA test was used to determine if there is a statistically significant difference in SWV amongst the various age groups with post-hoc Tukey-corrected pairwise multiple comparisons to highlight differences between each group and another.

The association between SWV and the muscle assessment variables was tested using Pearson’s correlation coefficients to understand how muscle stiffness correlates with strength and clinical characteristics. Moreover, the correlations with elderly-specific variables (ELSA frailly index, the SF-36 physical function and SF-36 general health) were evaluated. To further understand the underlying muscle stiffness process, multiple linear regression analysis was performed to evaluate if the independent variables (age, sex and BMI) are significantly associated with SWV.

## Results

### Participant information and muscle assessments

A total of 77 participants volunteered in this study, of which 26 young, 21 middle-aged and 30 elderly with a mean age (SD, range) of 28.1 (4.1, 20–35), 48.5 (5.2, 40–55) and 81.7 (4.1, 77–94), respectively. The descriptive statistics of the main characteristics are listed in Table 1. The elderly group had a mean (SD) frailty index, SF-36 physical function and SF-36 general health scores of 9.5 (3.3), 71.4 (21.9) and 70.0 (13.4), respectively. Muscle mass decreased on average by 13.1% in the elderly group compared to young.

**Table 1:** Main characteristics of the study participants

Characteristic	Young (*n* = 26)	Middle-aged (*n* = 21)	Elderly (*n* = 30)	*p* value^‡^
Sex	13 Females (50%)	15 Females (71%)	13 Females (56%)	0.32
Age (years)	28.1 (4.1)	48.5 (5.2)	81.7 (4.1)	< **0.001**
Males (years)	28.0 (4.0)	47.1 (5.2)	82.0 (4.2)	< **0.001**
Females (years)	28.3 (4.3)	49.1 (5.3)	81.4 (4.1)	< **0.001**
Height (cm)	169.0 (10.6)	167.2 (10.2)	161.2 (7.3)	**0.007**
Weight (kg)	71.3 (21.9)	71.7 (12.2)	75.5 (12.6)	0.57
BMI	24.5 (5.3)	25.6 (3.6)	28.8 (4.7)	**0.002**
Fat mass (kg)	16.1 (10.6)	18.9 (5.0)	26.7 (8.6)	< **0.001**
Muscle mass (kg)	53.3 (14.8)	51.0 (10.7)	46.3 (8.2)	0.08
Muscle mass index	18.3 (3.1)	17.7 (2.2)	17.7 (2.3)	0.69
Smoking	5 (23%)	7 (33%)	11 (37%)	0.18
Pack-years^a^	1.25 (4.3)	1.8 (1.3)	7.5 (16.5)	0.16
Drinking alcohol	13 (50%)	10 (43%)	21 (70%)	0.12
(units/week)^a^	5.0 (13)	6.0 (6.5)	5.0 (12)	0.58
VAS score (mm)^a^	6.0 (14)	6.5 (17)	10.0 (25)	0.25

^a^Median and interquartile range

^‡^*p* values significant at 95% are highlighted in bold

The one-way ANOVA results of the muscle assessments in Table 2 showed a significant difference amongst the three age groups (*p* < 0.05). Further analysis via post hoc multiple comparisons revealed that the significant differences only existed between the elderly group and the two younger groups. However, the knee extension torque and power were the only tests that showed significant differences between all groups (*p* < 0.001). The results for the subcomponents of the ETGUG test were similar to each other; hence, only the total ETGUG test time was reported. The elderly group were approximately 4 s slower in the ETGUG test. They had a significantly lower grip strength of 26.3 kg compared to 36.7 kg and 35.4 kg for young and middle-aged, respectively. On average, the young and middle-aged performed 5–7 chair stands in 30 s more than the elderly (*p* < 0.001).

**Table 2:** Results of the muscle assessments for the three age groups

Characteristic	Young^(a)^		Middle aged^(b)^		Elderly^(c)^		*p* value	Post hoc^a^
	Mean (SD)	95% CI	Mean (SD)	95% CI	Mean^a^	95% CI		
ETGUGT, total time-(s)	16.1 (1.4)	15.6–16.7	16.4 (3.1)	15.0–17.8	20.6 (3.6)	19.3–22.0	< **0.001**	a, b < c
30-s chair sit-to-stands	20.0 (5.8)	17.7–22.4	17.9 (3.1)	15.3–20.6	12.3 (4.4)	10.7–14.0	< **0.001**	a, b < c
Handgrip strength-(kg)	36.7 (10.8)	32.3–41.2	35.4 (9.0)	31.3–39.5	26.3 (10.6)	22.4–30.3	**0.001**	a, b < c
Knee extension torque-(Nm/kg)	1.9 (0.6)	1.7–2.2	1.5 (0.5)	1.2–1.7	0.8 (0.3)	0.7–0.9	< **0.001**	a > b > c
Knee flexion torque-(Nm/kg)	1.0 (0.3)	0.9–1.1	0.9 (0.2)	0.7–1.0	0.5 (0.2)	0.4–0.5	< **0.001**	a, b > c
Knee extension power-(W/kg)	1.2 (0.4)	1.0–1.4	0.9 (0.3)	0.7–1.0	0.4 (0.2)	0.4–0.5	< **0.001**	a > b > c
Knee flexion power-(W/kg)	0.6 (0.2)	0.5–0.7	0.5 (0.1)	0.4–0.6	0.3 (0.1)	0.2–0.3	< **0.001**	a, b > c

^a^Result of the post hoc Tukey-corrected multiple comparisons

### Shear wave elastography

**Fig. 2:**
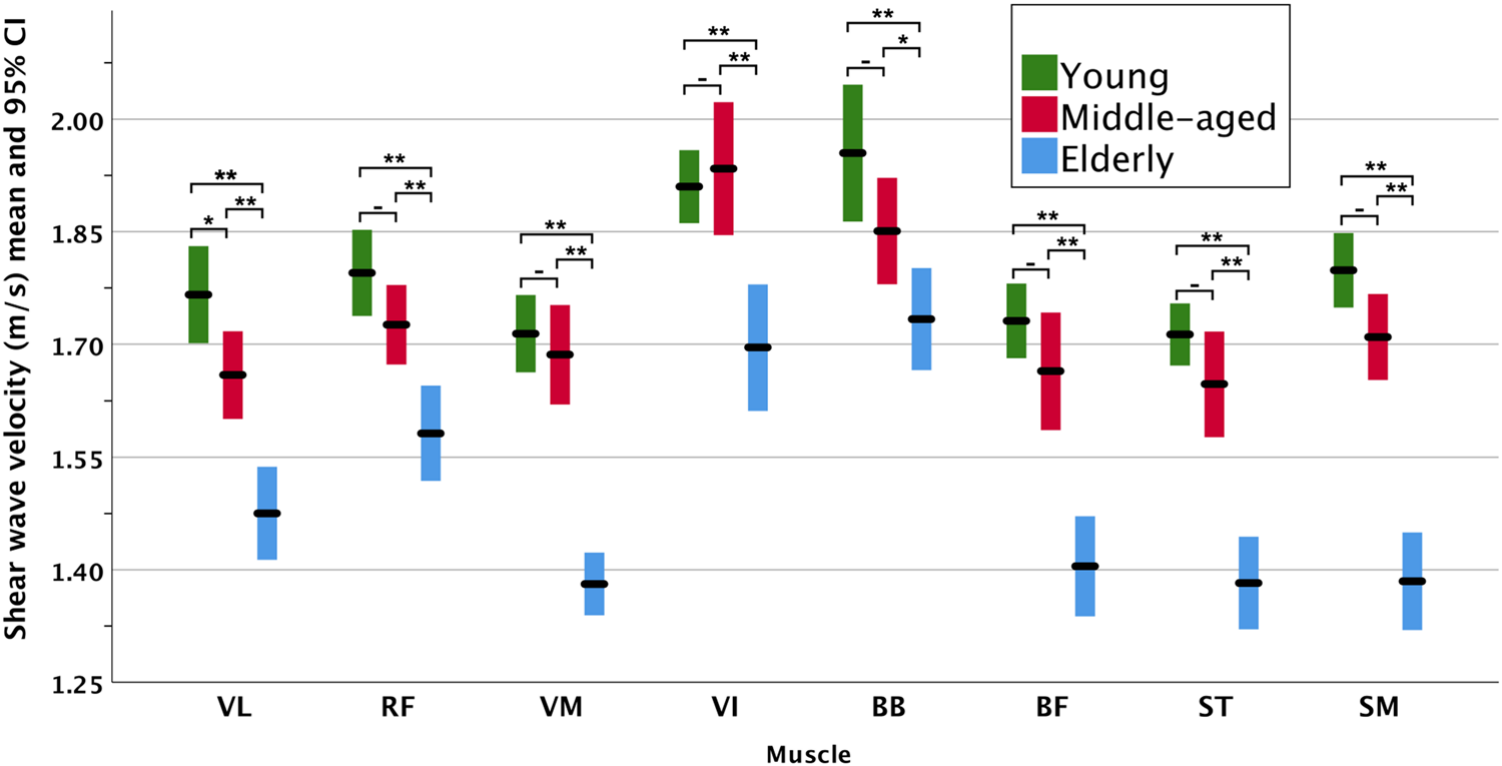
Clustered error bars for the mean muscle shear wave velocity in the relaxed resting position. The asterisks indicate significance at 0.05 level (*) and 0.01 (**). The hyphens (-) indicate lack of statistical significance (*p* > 0.05)

**Fig. 3:**
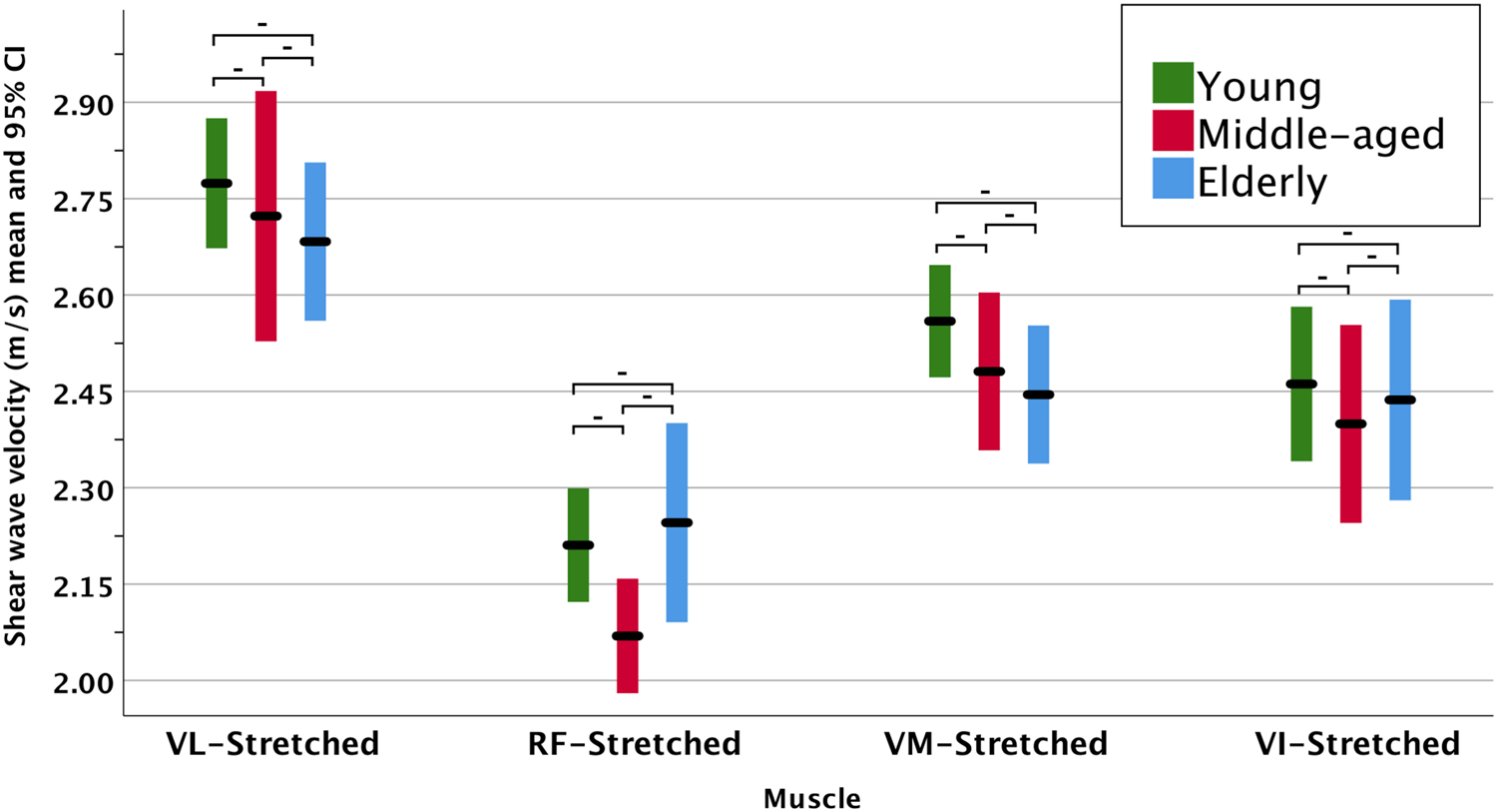
Clustered error bars for the mean muscle shear wave velocity during in the passively stretched position. The hyphens (-) above the bars indicate lack of statistical significance (*p* > 0.05)

The SWE measurements listed in Table 3 identified significant differences (*p* < 0.001) during the resting position for all the tested lower limb muscles and the BB. The SWV was higher in the passively stretched position in the quadriceps. However, it did not result in significant differences amongst the age groups. The results can be better appreciated graphically in the clustered error bars in Fig. 2 during the resting position and in Fig. 3 during the passive stretching position.

**Table 3:** Muscle shear wave velocity in the healthy young, middle-aged and elderly participants

Muscle	Young^(a)^		Middle aged^(b)^		Elderly^(c)^		*p* value^*^	Post hoc^a^
	Mean (SD)	95% CI	Mean (SD)	95% CI	Mean (SD)	95% CI		
Vastus lateralis-(VL)	1.77 (0.15)	1.70–1.83	1.64 (0.12)	1.59, 1.70	1.48 (0.16)	1.42–1.53	< **0.001**	a > b > c
Passively stretched	2.77 (0.24)	2.68–2.87	2.72 (0.42)	2.53, 2.92	2.68 (0.31)	2.56–2.80	0.54	–
Rectus femoris-(RF)	1.80 (0.14)	1.74–1.85	1.72 (0.11)	1.67, 1.77	1.58 (0.16)	1.52–1.64	< **0.001**	a, b > c
Passively stretched	2.21 (0.21)	2.13–2.30	2.07 (0.18)	1.99, 2.16	2.25 (0.41)	2.09–2.40	0.18	–
Vastus Medialis-(VM)	1.71 (0.12)	1.67–1.76	1.68 (0.14)	1.62, 1.74	1.38 (0.10)	1.34–1.42	< **0.001**	a, b > c
Passively stretched	2.56 (0.20)	2.48–2.64	2.50 (0.26)	2.38, 2.62	2.45 (0.28)	2.34–2.55	0.21	–
Vastus Intermedius-(VI)	1.91 (0.11)	1.86–1.96	1.92 (0.19)	1.83, 2.01	1.70 (0.22)	1.61–1.78	< **0.001**	a, b > c
Passively stretched	2.46 (0.28)	2.34–2.58	2.39 (0.33)	2.24, 2.54	2.44 (0.40)	2.28–2.59	0.72	–
Biceps Brachii-(BB)	1.95 (0.22)	1.87–2.04	1.85 (0.15)	1.78, 1.92	1.73 (0.18)	1.67–1.80	< **0.001**	a, b > c
Biceps Femoris-(BF)	1.73 (0.12)	1.68–1.78	1.64 (0.14)	1.58, 1.70	1.40 (0.17)	1.34–1.47	< **0.001**	a, b > c
Semitendinosus-(ST)	1.71 (0.10)	1.67–1.75	1.64 (0.14)	1.57, 1.70	1.38 (0.16)	1.32–1.44	< **0.001**	a, b > c
Semimembranosus-(SM)	1.80 (0.12)	1.75–1.85	1.70 (0.12)	1.65, 1.76	1.38 (0.17)	1.32–1.45	< **0.001**	a, b > c

*Significant one-way ANOVA *p* values at 95% are highlighted in bold

^a^Result of the post hoc Tukey-corrected multiple comparisons

The mean SWV difference between the middle-aged and young groups over all muscles was − 4.1% ranging from 0.5 to − 7.3%. As for the elderly and middle-aged, the mean difference was − 12.9% and ranged from − 6.5 to − 18.8%. However, the highest mean difference of − 16.5% was observed between the elderly and young groups with the differences amongst the muscles ranging from − 11.0 to − 23.3%. The VI and BB exhibited the smallest differences, whereas the VM and SM were the highest.

**Fig. 4:**
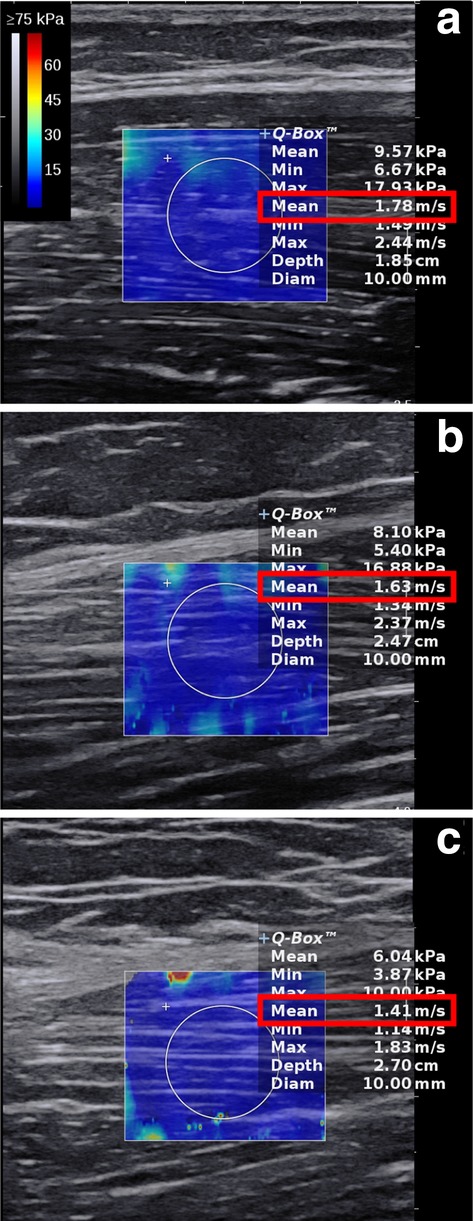
Shear wave elastography examples from **a** young (22 years), **b** middle-aged (55 years) and **c** elderly (80 years) participants

Despite the gradual decreasing SWV across almost all muscle, the post hoc analysis revealed that only the VL had a significantly decreasing SWV between all three age groups. The other muscles were only significantly lower in the elderly participants (*p* < 0.001) compared to the other two groups. SWE examples are shown in Fig. 4.

The correlations between SWV and the main clinical and muscle assessment variables are listed in Table 4. On average, there were significant moderate correlations between SWV and the tested variables. Age had a significant negative correlation ranging from − 0.450 to − 0.822 for the BB and SM, respectively (*p* < 0.001). The muscle mass index, however, did not correlate with any of the muscle SWV (*r* < 0.15; *p* > 0.05). The strongest correlations were found for the Biodex knee extension/flexion exercise. Overall, the VL from the quadriceps and SM from the hamstrings exhibited the strongest correlations.

**Table 4:** Muscle shear wave velocity correlations with clinical and muscle test variables for all participants

	VL	RF	VM	VI	BF	ST	SM	BB
Age	− 0.680^b^	− 0.568^b^	− 0.770^b^	− 0.464^b^	− 0.744^b^	− 0.778^b^	− 0.822^b^	− 0.450^b^
BMI	− 0.346^b^	− 0.323^b^	− 0.210	− 0.323^b^	− 0.315^b^	− 0.316^b^	− 0.427^b^	− 0.250^a^
Fat mass	− 0.453^b^	− 0.421^b^	− 0.385^b^	− 0.392^b^	− 0.474^b^	− 0.414^b^	− 0.542^b^	− 0.330^b^
Muscle mass	0.216	0.146	0.276^a^	0.226	0.266^a^	0.231	0.316^b^	0.189
ETGUGT, total time	− 0.386^b^	− 0.288^a^	− 0.367^b^	− 0.380^b^	− 0.338^b^	− 0.366^b^	− 0.560^b^	− 0.198
30-s Chair stand test	0.354^b^	0.294^b^	0.482^b^	0.261^a^	0.373^b^	0.365^b^	0.522^b^	0.267^a^
Handgrip Strength	0.285^a^	0.256^a^	0.343^b^	0.293^a^	0.344^b^	0.297^b^	0.436^b^	0.287^a^
Knee extension torque	0.587^b^	0.454^b^	0.636^b^	0.356^b^	0.547^b^	0.542^b^	0.640^b^	0.443^b^
Knee flexion torque	0.540^b^	0.405^b^	0.603^b^	0.385^b^	0.497^b^	0.491^b^	0.641^b^	0.422^b^
Knee flexion power	0.579^b^	0.447^b^	0.613^b^	0.350^b^	0.535^b^	0.501^b^	0.623^b^	0.456^b^
Knee extension power	0.540^b^	0.439^b^	0.600^b^	0.333^b^	0.489^b^	0.479^b^	0.640^b^	0.422^b^

^a^Correlation is significant at the 0.05 level (2-tailed)

^b^Correlation is significant at the 0.01 level (2-tailed)

In the elderly group, there was a significant negative correlation between SWV and the ELSA frailty index in the RF, VI and SM with coefficients (*p* values) of − 0.386 (0.035), − 0.470 (0.009) and − 0.412 (0.024), respectively. However, there were no significant correlations with muscle mass or muscle mass index.

The multiple linear regression determined that neither sex or BMI explained a significant variation in SWV in all muscles. In contrast, age was consistently a significant predictor of SWV in the regression model. The adjusted *R*^2^ for the VL, RF, VM, VI, BB, BF, ST and SM were 0.455, 0.314, 0.587, 0.205, 0.191, 0.547, 0.600 and 0.671, respectively. In other words, 45.5% of the variance in SWV is explained by age in the VL muscle as an example. The results of the two models on the investigated muscles are included in Supplementary Table 1.

## Discussion

The primary aim of this study was to investigate the effect of ageing on muscle stiffness using SWE. To our knowledge, this is the first study to incorporate strength and functional correlations to substantiate their associations with muscle stiffness throughout an adult age-span. We systematically scanned all quadriceps and hamstring muscles, recommended in a previous study and not been simultaneously investigated before [14]. Additionally, the majority of prior work have not included elderly subjects above 75, which has previously been recommended [15]. We achieved this by recruiting healthy elderly participants up to 94 years.

The results demonstrated a gradual reduction in resting muscle stiffness throughout adulthood with a significant decline most notable in the elderly (> 75 years) group. This was true for all the tested muscles, which display different fibre arrangements and functions. These findings support the hypothesis that known age-related histological features of muscle such as myosteatosis, variability in myofiber size and impaired connective tissue may promote the loss of normal muscle stiffness. This was also proposed previously by the work of Rodrigues & Rodrigues Junior [9], consistent with the elastic fibre system in the muscle extracellular matrix starting to lose its resistance property and becoming softer with ageing.

In contrast, this study has not confirmed previous in-vitro results by Ochala et al. [16], which reported increased muscle stiffness with ageing due to increased collagen concentrations. Brown et al. [7], in contrast, reported that muscle stiffness is not significantly different between young and old rats. However, these studies used a fundamentally different biomechanical approach (testing the passive tension–length relationship) for evaluating muscle stiffness. There is also a lack of consensus in the SWE literature as to whether muscle stiffness decreases [14, 17], increases [18] or does not change [19] with ageing. Akagi et al. [14] reported a significant reduction of 17% in the RF stiffness, decreasing from 3.4 kPa in the young to 2.8 kPa in the elderly (*p* = < 0.001). Similarly, Yoshida et al. [17] found a comparable decline in muscle stiffness of approximately 9% in the gastrocnemius. These percentages are similar to the observed average range of reduction in this study.

Studies using SWE [19] and magnetic resonance elastography [20] confirm the observed decreasing trend with a lack of statistical significance between young and middle-aged groups. The muscle ageing process does not appear to start until the sixth decade when declines in muscle strength and mass emerge [21]. Significant declines take place above 75 years as risks of fall and frailty increase [22]. This probably explains the overall lack of difference between young and middle-aged, except in VL.

It is interesting that muscle stiffness during passive stretching is preserved in older age, which was also observed in a previous study on the VI muscle [19]. The mechanism behind this remains unclear. Considering that all of the recruited participants were healthy, it may be speculated that passive muscle stiffness is preserved to maintain normal muscle contractility and function.

The secondary aim of this study was to elucidate the relationship of muscle stiffness to muscle mass and strength, the principal components of sarcopenia and dynapenia, respectively. There was a stronger correlation with fat mass compared to muscle mass, which correlated poorly with just two of the scanned muscles. On the other hand, weaker muscles and worse physical performance were associated with decreased muscle stiffness. Considering that the increase in muscle compliance is related to a decrease in muscle strength [23], the observed loss in muscle stiffness in the elderly group may explain the positive correlation with low muscle strength.

The implications of this study findings are important. The decreased muscle stiffness in the elderly subject and its correlation with reduced strength could compromise the delivery of tensile muscle power of the muscle fibres to the tendon. This may suggest an increased exposure risk of muscle fibres to rupture or deformation, consequently increasing rates of physical frailty. Hence, SWE could be a useful tool for evaluating and screening elderly subjects at risk of sarcopenia and frailty. Besides, the higher average reduction between elderly and young in muscle stiffness (16.5%) compared to muscle mass (13.1%) may speculate a better role of SWE in predicting earlier muscle changes associated with ageing. However, further research is required to establish these roles. In terms of future study design, the results highlight the importance of adjusting for age in future muscle SWE studies, especially when the cohort involves a wide age range.

The results hold promise for SWE in the field of elderly care medicine for highlighting the impact of ageing on muscle stiffness. Future studies should aim to compare muscle stiffness in sarcopenic and non-sarcopenic subjects to elucidate the value of SWE for assessing muscle quality. Currently, abnormal muscle stiffness is not a risk factor for falls in the elderly population; researching this could yield valuable evidence. Future studies are also encouraged to control for physical activity to strengthen the relationship between muscle stiffness and age.

The findings in this study are subject to several limitations. First, the three age groups were not matched in size. Second, the study did not evaluate the passive stretching on the BB or hamstring muscle to avoid long scanning time. Moreover, the study design does not represent the complete adulthood age-span since it lacked information on younger subjects 56–74 years. Finally, inter-operator reproducibility was not verified.

In conclusion, ageing was associated with a decline in both lower and upper limb skeletal resting muscle stiffness as measured by SWE. The greatest reduction in muscle stiffness was found in elderly participants > 75 years. The decline correlated stronger with lower muscle strength than muscle mass. Sex and BMI did not influence muscle stiffness. The age-related changes in muscle stiffness may contribute to our understanding of the development of musculoskeletal disorders with age. The results highlight the needs for further research to investigate if decreased muscle stiffness can predict fall risk in elderly individuals. Overall, the results emphasise the importance of controlling for the age variable in muscle SWE studies.

## Electronic supplementary material

Below is the link to the electronic supplementary material.

Supplementary material 1 (DOCX 126 KB)
