# Supplementary material for: The effect of ageing on shear wave elastography muscle stiffness in adults
Source: Aging Clin Exp Res. 2019 Feb 14;31(12):1755–63. doi: 10.1007/s40520-019-01139-0 (PMC6825644; doi:10.1007/s40520-019-01139-0)
Supplement: Supplementary file 1 — Supplementary material 1 (DOCX 126 KB) [file 40520_2019_1139_MOESM1_ESM.docx]

## The effect of ageing on shear wave elastography muscle stiffness in adults

## Authors:

Abdulrahman M. Alfuraih ^1,2,3^ (ORICD: 0000-0002-4655-7248)

Ai Lyn Tan ^2,3^ (ORICD: 0000-0002-9158-7243)

Philip O’Connor ^3^

Paul Emery ^2,3^

Richard J. Wakefield ^2,3^ (ORICD: 0000-0001-5352-8683)

## Affiliations:

^1^Radiology and Medical Imaging Department, College of Applied Medical Sciences, Prince Sattam bin Abdulaziz University, Kharj, Saudi Arabia.

^2^Leeds Institute of Rheumatic and Musculoskeletal Medicine, Chapel Allerton Hospital, University of Leeds, United Kingdom.

^3^NIHR Leeds Biomedical Research Centre, Leeds Teaching Hospitals NHS Trust, Leeds, United Kingdom.

## Corresponding author:

Abdulrahman M. Alfuraih

LIRMM, 2nd Floor,

Chapel Allerton Hospital,

Chapeltown Road, Leeds, LS7 4SA, UK.

Phone: +44 113 392 4854

E-mail: mt14ama@leeds.ac.uk

**Supplementary figure.**


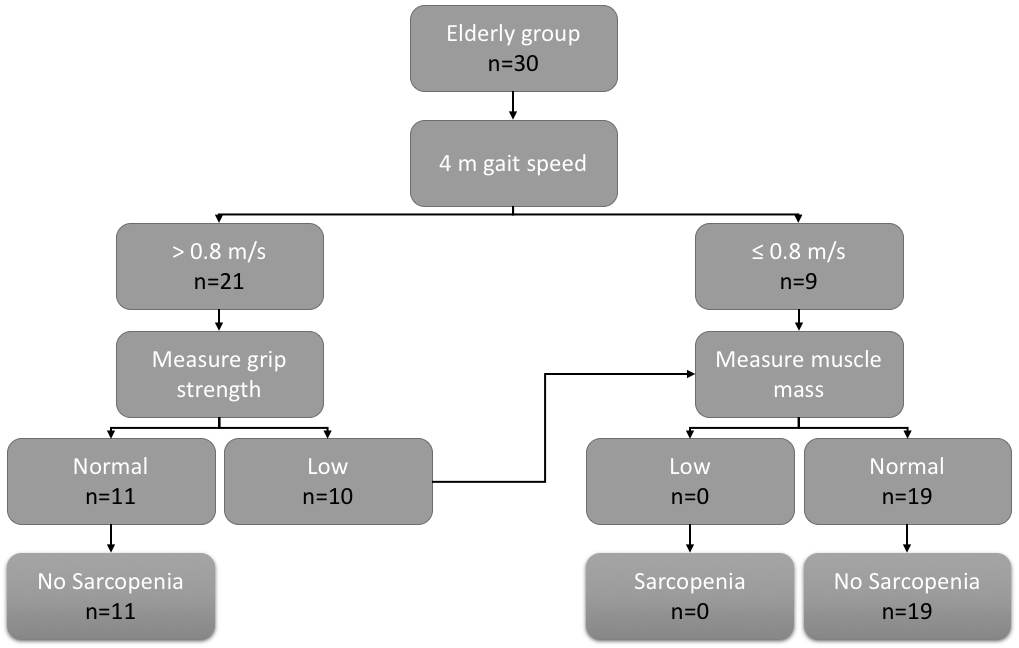


**Supplementary Fig.1** Flowchart of sarcopenia assessment in the elderly age group.

**Supplementary table.**

Supplementary table 1. Multiple regression predicting shear wave velocity from age, sex and body mass index.

|  | **Vastus Lateralis** | | | | | |
| --- | --- | --- | --- | --- | --- | --- |
|  | **Model 1** | | | **Model 2** | | |
| **Variable** | **B** | **β** | **Sig.** | **B** | **β** | **Sig.** |
| Constant | .662 |  |  | .771 |  |  |
| Age | -.003 | -.680 | **<.001**** | -.003 | -.628 | **<.001**** |
| Sex |  |  |  | -.025 | -.106 | .219 |
| BMI |  |  |  | -.003 | -.131 | .160 |
| R^2^ | .463 | |  | .484 | |  |
| △R^2^ |  | |  | .021 | | .160 |
|  | **Rectus Femoris** | | | | | |
|  | **Model 1** | | | **Model 2** | | |
| **Variable** | **B** | **β** | **Sig.** | **B** | **β** | **Sig.** |
| Constant | .653 |  |  | .732 |  |  |
| Age | -.002 | -.568 | **<.001**** | -.002 | -.514 | **<.001**** |
| Sex |  |  |  | -.010 | -.050 | .606 |
| BMI |  |  |  | -.003 | -.141 | .179 |
| R^2^ | .323 | |  | .340 | |  |
| △R^2^ |  | |  | .017 | | .179 |
|  | **Vastus Medialis** | | | | | |
|  | **Model 1** | | | **Model 2** | | |
| **Variable** | **B** | **β** | **Sig.** | **B** | **β** | **Sig.** |
| Constant | .672 |  |  | .606 |  |  |
| Age | -.004 | -.770 | **<.001**** | -.004 | -.805 | **<.001**** |
| Sex |  |  |  | .009 | .035 | .643 |
| BMI |  |  |  | .002 | .092 | .261 |
| R^2^ | .593 | |  | .600 | |  |
| △R^2^ |  | |  | .007 | | .261 |
|  | **Vastus Intermedius** | | | | | |
|  | **Model 1** | | | **Model 2** | | |
| **Variable** | **B** | **β** | **Sig.** | **B** | **β** | **Sig.** |
| Constant | .725 |  |  | .911 |  |  |
| Age | -.002 | -.464 | **<.001**** | -.002 | -.378 | **<.001**** |
| Sex |  |  |  | -.047 | -.196 | .056 |
| BMI |  |  |  | -.005 | -.214 | .053 |
| R^2^ | .215 | |  | .279 | |  |
| △R^2^ |  | |  | .064 | | .053 |
|  | **Biceps Brachii** | | | | | |
|  | **Model 1** | | | **Model 2** | | |
| **Variable** | **B** | **β** | **Sig.** | **B** | **β** | **Sig.** |
| Constant | .719 |  |  | .770 |  |  |
| Age | -.002 | -.450 | **<.001**** | -.002 | -.413 | **<.001**** |
| Sex |  |  |  | -.001 | -.005 | .965 |
| BMI |  |  |  | -.002 | -.099 | .387 |
| R^2^ | .202 | |  | .210 | |  |
| △R^2^ |  | |  | .008 | | .387 |
|  | **Biceps Femoris** | | | | | |
|  | **Model 1** | | | **Model 2** | | |
| **Variable** | **B** | **β** | **Sig.** | **B** | **β** | **Sig.** |
| Constant | .676 |  |  | .750 |  |  |
| Age | -.004 | -.744 | **<.001**** | -.004 | -.717 | **<.001**** |
| Sex |  |  |  | -.023 | -.087 | .275 |
| BMI |  |  |  | -.002 | -.064 | .455 |
| R^2^ | .553 | |  | .562 | |  |
| △R^2^ |  | |  | .009 | | .455 |
|  | **Semitendinosus** | | | | | |
|  | **Model 1** | | | **Model 2** | | |
| **Variable** | **B** | **β** | **Sig.** | **B** | **β** | **Sig.** |
| Constant | .672 |  |  | .682 |  |  |
| Age | -.004 | -.778 | **<.001**** | -.004 | -.768 | **<.001**** |
| Sex |  |  |  | .005 | .019 | .803 |
| BMI |  |  |  | -.001 | -.031 | .707 |
| R^2^ | .606 | |  | .607 | |  |
| △R^2^ |  | |  | .001 | | .707 |
|  | **Semimembranosus** | | | | | |
|  | **Model 1** | | | **Model 2** | | |
| **Variable** | **B** | **β** | **Sig.** | **B** | **β** | **Sig.** |
| Constant | .749 |  |  | .844 |  |  |
| Age | -.005 | -.822 | **<.001**** | -.005 | -.759 | **<.001**** |
| Sex |  |  |  | -.005 | .087 | .186 |
| BMI |  |  |  | .026 | -.161 | **.025*** |
| R^2^ | .675 | |  | .701 | |  |
| △R^2^ |  | |  | .026 | | .052 |

B= unstandardized coefficient. β=standardised coefficient. R^2^= coefficient of determination. **p* < 0.05. ***p* < 0.01.
